# Daphnetin Improves Neuropathic Pain by Inhibiting the Expression of Chemokines and Inflammatory Factors in the Spinal Cord and Interfering with Glial Cell Polarization

**DOI:** 10.3390/ph16020243

**Published:** 2023-02-06

**Authors:** Tianrui Zhang, Wulin Liang, Mingqian Zhang, Shuang Cui, Xiyan Huang, Wenjing Ou, Rikang Huang, Jiahui Gao, Zhanhong Jia, Shuofeng Zhang

**Affiliations:** Department of Pharmacology of Traditional Chinese Medicine, College of Chinese Medicine, Beijing University of Chinese Medicine, Beijing 102488, China

**Keywords:** neuropathic pain, daphnetin, chemokines, glial cells, polarization

## Abstract

Neuropathic pain (NP) is a common pain disease that seriously affects the quality of life and physical and mental health of patients. Daphnetin is extracted from the *Daphne giraldii* Nitsche and has the structure of 7,8-dihydroxy coumarin. As a natural product, daphnetin displays a wide range of pharmacological activities, such as analgesia and anti-inflammatory activities, but whether it is able to improve NP through anti-inflammatory effects is unknown. Therefore, this paper intends to investigate the mechanism of daphnetin in improving NP rats affected by the intrathecal injection of tumor necrosis factor-α (TNF-α) from the perspective of anti-inflammation. Our results showed that daphnetin significantly improved hyperalgesia in NP rats. Daphnetin inhibited the activation and polarization of glial cells and neurons in the spinal cord of NP rats and reduced the expression of mRNA and protein of inflammatory factors and chemokine pairs in the spinal cord. Daphnetin inhibited the polarization of human microglia cell 3 (HMC3) cells and human glioma cells (U251) cells toward M1 microglia and A1 astrocytes, respectively, and induced the conversion of M1 microglia and A1 astrocytes to M2 microglia and A2 astrocytes, respectively. In conclusion, daphnetin ameliorates NP by inhibiting the expression of inflammatory factors and chemokines and the polarization of glial cells in the spinal cord of NP rats. This study provides a theoretical basis for the treatment of NP with daphnetin to expand the clinical application of daphnetin.

## 1. Introduction

Neuropathic pain (NP) refers to pain caused by the injury or disease of the somatosensory system, which can be caused by peripheral nerve trauma or spinal cord and brain injury [1]. Depending on the site of nerve injury, NP can be divided into peripheral NP (diabetic neuralgia, cancer pain, trigeminal neuralgia, and postherpetic neuralgia, etc.) and central NP (spinal cord injury, multiple sclerosis, post-stroke pain, and Parkinson’s pain, etc.) [2]. The main clinical manifestations of NP are hyperalgesia, allodynia, and spontaneous pain, which can seriously affect the quality of life and physical and mental health of patients, as it is often accompanied by sleep disorders, anxiety, depression, etc. [3]. Currently, the incidence of NP in the general population is between 7 and 10% and will increase further as the population ages, the incidence of diabetes increases, and the pain associated with cancer increases [2]. Therefore, it is of great significance to find a treatment for NP.

The pathogenesis of NP is complex and involves numerous pathological factors that affect abnormal changes in normal sensory signals at the peripheral nerve, spinal cord, and brain levels, summarized in relation to peripheral nerve sensitization, central sensitization, and downstream inhibitory changes [1]. These key pathological mechanisms are interdependent and contribute to each other, ultimately leading to increased excitability and the decreased inhibition of neurons in the spinal cord and brain nociceptive system, resulting in abnormal pain. Moreover, central sensitization plays a key role in NP [4]. Central sensitization is a plastic change in the nervous system associated with inflammatory factor infiltration, de-inhibition, and long-duration enhancement of injurious neurons in the dorsal horn of the spinal cord [4]. Chemokines are small molecular proteins with cell recruitment functions, which participate in a variety of physiological and pathological processes [5]. Numerous studies have demonstrated that chemokines and their receptors are involved in the initiation and maintenance of NP [6,7]. In the central nervous system, chemokines are often expressed in microglia, astrocytes, and neurons. Nerve injury induces glial cell activation, which causes large numbers of chemokines to enhance neuronal excitability, and chemokines produced by neurons are also able to amplify the glial cell-mediated inflammatory response and thereby participate in the regulation of central sensitization [8]. Inhibiting the production of chemokines and chemokine receptors or blocking the function of chemokine receptors can effectively alleviate NP.

In recent years, the role of microglia in NP has been more intensively studied. Microglia can either be classically activated (M1) and alternative activated (M2). M1 microglia exert pro-inflammatory effects by releasing inflammatory factors such as interleukin-1β (IL-1β), interleukin-6 (IL-6), and tumor necrosis factor α (TNF-α), while M2 microglia exert anti-inflammatory effects by releasing interleukin-4 (IL-4) and interleukin-10 (IL-10) [9]. Under normal conditions, microglia remain stable and protective of neurons. In the NP model induced by peripheral nerve injury, microglia activation is accompanied by an imbalance in polarization, and as nerve injury progresses, microglia in the spinal cord tend towards a single expression of the M1 type [10]. Activated M1 microglia express a variety of molecules, such as surface receptors, cytokines, and transcription factors, while activating multiple signaling pathways to enhance neuronal excitability [11]. The study shows that inhibiting the activation of M1 microglia and promoting the activation of M2 microglia can inhibit NP [12]. The other major glial cell, the astrocyte, has also been shown to have two distinct phenotypes upon activation: A1 and A2 astrocytes [13]. A1 astrocytes highly express a variety of classical complement cascade response genes, which damage neuronal cells and oligodendrocytes and exhibit neurotoxic effects, while A2 astrocytes express many neurotrophic factors, participate in synapse formation, promote central nervous system repair, and exert neuroprotective effects [14]. A1 and A2 reactive astrocytes have pro-inflammatory and anti-inflammatory effects, respectively, and promoting the conversion of A2 reactive astrocytes to the A1 type increases neuroinflammation [15]. The regulation of A1 astrocyte generation or phenotypic transition has the potential to be a new target for NP control.

**Figure 1:**
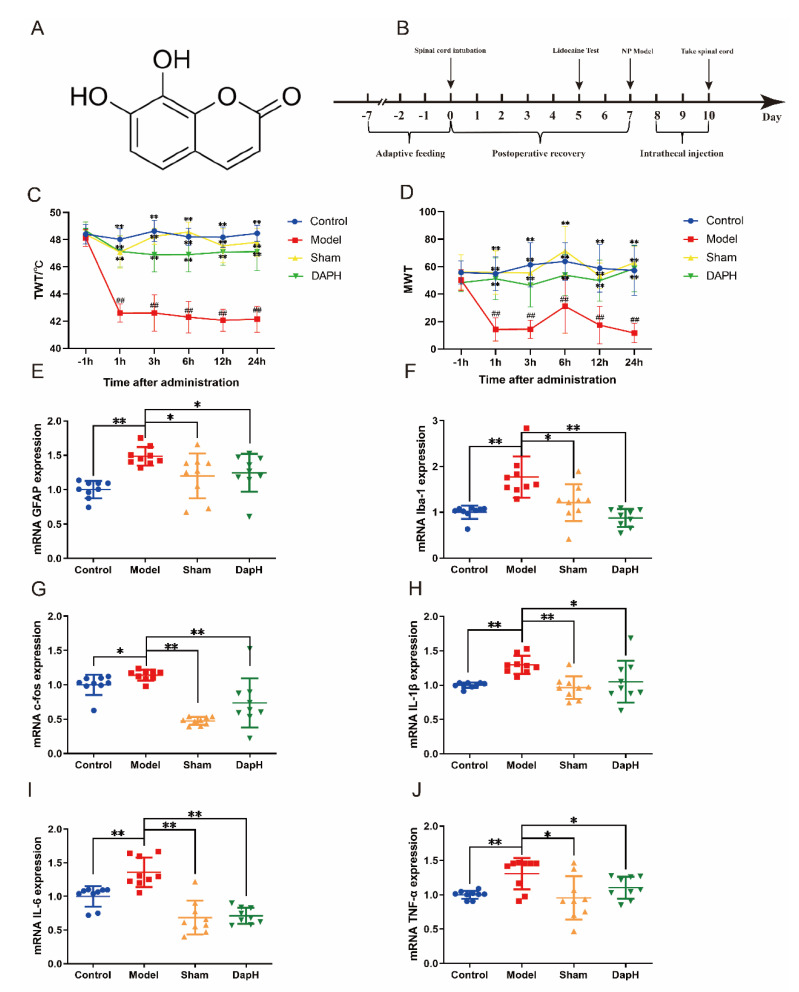
Effect of daphnetin on hyperalgesia in NP rats. (**A**) Structural formula of daphnetin. (**B**) Experimental flowchart. (**C**) Changes in TWT in NP rats (*n* = 9). (**D**) Changes in MWT in NP rats (*n* = 9). (**E**) mRNA expression of GFAP, a marker of astrocyte activation in the spinal cord (*n* = 3). (**F**) mRNA expression of Iba−1, a marker of microglia activation in the spinal cord (*n* = 3). (**G**) mRNA expression of c−fos, a marker of neuronal activation in the spinal cord (*n* = 3). (**H**–**J**) mRNA expression of inflammatory factors in the spinal cord (*n* = 3). * *p* < 0.05, ** *p* < 0.01 vs. model group (ANOVA); ## *p* < 0.01 vs. before molding (Student’s *t*-test).

Daphnetin (Dap), structured as 7,8-dihydroxycoumarin, as detailed in Figure 1A, is the main active ingredient extracted from *Daphne giraldii* Nitsche, a plant of the family daphne, with a wealth of biological activities, including anti-inflammatory, antioxidant, neuroprotective, analgesic, and anticancer activities [16]. Daphnetin significantly inhibits the development of adjuvant arthritis by inhibiting the production of pro-inflammatory cytokines [17]. Tu et al. [18] also reported that daphnetin attenuated inflammatory and pathological changes in rat joint tissues in collagenous arthritis, as well as synovial hyperplasia and chondrocyte degeneration, suggesting an ameliorative effect of daphnetin on acute pain in arthritis. However, the effect of daphnetin on NP and its mechanism are not clear. Based on this, this study was conducted to explore the role of daphnetin in NP and related molecular mechanisms by using the intrathecal injection of TNF-α to induce NP production in rats, combined with in vitro experiments.

## 2. Results

### 2.1. Daphnetin Improves Hyperalgesia in NP Rats

TNF-α is widely involved in the development of NP and is highly expressed in the spinal cord in a variety of animal models of pain [19]. After the surgery, we observed no rats with paralysis caused by spinal cord injury, and the movement of the rats was not affected. We maintained a 7-day recovery period after subdural intubation to ensure the healthy state of the rats before establishing the NP model (Figure 1B). In this experiment, after the injection of TNF-α in the subdural space, the rats showed significant pain in their lower extremities, manifested by over-sensitivity to thermal and mechanical stimuli, and a significant decrease in thermal withdrawal threshold (TWT) and mechanical withdrawal threshold (MWT). After the intrathecal injection of daphnetin, daphnetin was effective in improving nociceptive sensitivity in NP rats, as evidenced by the modulation of TWT and MWT. By testing multiple times after administration, we found that the analgesic effect of a single injection of daphnetin lasted for more than 24 h (see Figure 1B,C).

### 2.2. Daphnetin Inhibits the Activation of Glial Cells and Neurons in the Spinal Cord of NP Rats

The intrathecal injection of TNF-α in rats can activate astrocytes in the spinal cord and induce central sensitization to produce NP [20]. The transient activation of c-Jun N-terminal protein kinase (JNK) via TNF receptor 1 induces astrocytes to polarize toward the A1 type and produce large amounts of inflammatory factors [21]. In this experiment, we tracked the changes in glial fibrillary acidic protein (GFAP) mRNA in astrocytes in the rats’ spinal cords using quantitative real-time polymerase chain reaction (qRT-PCR) and found that GFAP was significantly elevated in the model group, and its expression was suppressed after the intrathecal injection of daphnetin. In addition, we examined the mRNA expression of the ionized calcium-binding adapter molecule-1 (Iba-1), a marker of microglia activation, and c-fos, a marker of neuronal activation, in the spinal cord of NP rats, and found that the mRNA expression of both Iba-1 and c-fos was upregulated in the spinal cord of rats in the model group. Meanwhile, the mRNA expression of inflammatory factors IL-1β, IL-6, and TNF-α was upregulated in the spinal cord of rats after the intrathecal injection of TNF-α, and the above changes showed significant improvement after the intrathecal injection of daphnetin. Following the intrathecal injection of TNF-α, the activation of the triad of spinal microglia, astrocytes, and neurons secreting inflammatory factors induced central sensitization, which in turn led to the production of NP in rats (see Figure 1D–I).

### 2.3. Daphnetin Inhibits the Polarization of A1 Astrocytes in the Spinal Cord of NP Rats

**Figure 2:**
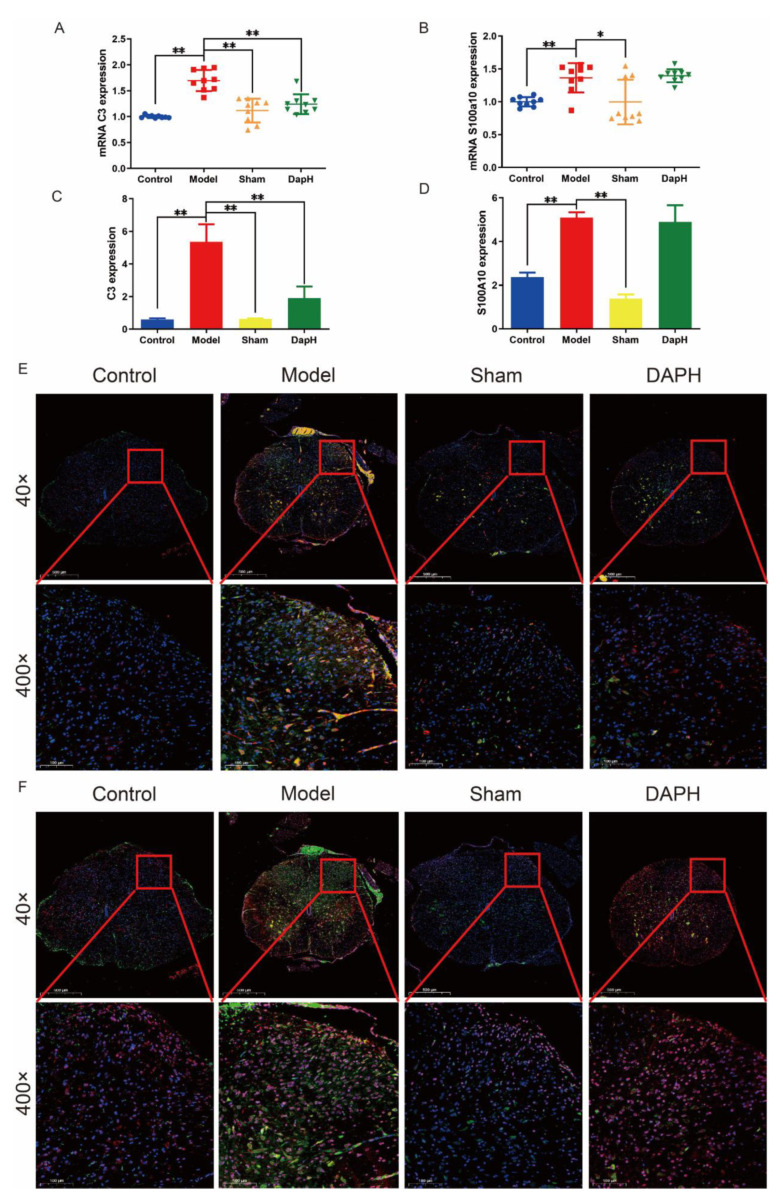
Effect of daphnetin on astrocyte polarization in the spinal cord of NP rats. (**A**) mRNA expression of A1 astrocyte markers in the spinal cord (*n* = 3). (**B**) mRNA expression of A2 astrocyte markers in the spinal cord (*n* = 3). (**C**) Immunofluorescence intensity of C3 in immunofluorescence sections (*n* = 3). (**D**) Immunofluorescence intensity of S100A10 in immunofluorescence sections (*n* = 3). (**E**) Immunofluorescence double staining of C3 (red) and GFAP (green) in the spinal cord of NP rats (*n* = 3). (**F**) Immunofluorescence double staining of S100A10 (red) and GFAP (green) in the spinal cord of NP rats (*n* = 3). * *p* < 0.05, ** *p* < 0.01. The blue circles, red squares, green and yellow triangles refer to the individual data of the Control, Model, Sham and DAPH groups, respectively.

The literature has demonstrated the neuroprotective and neurotoxic effects of reactive astrocytes in neurodegenerative diseases and spinal cord injury [22]. A1 astrocytes may secrete molecules such as pro-inflammatory factors and chemokines that are involved in the process of pain and contribute to the development of chronic pain; conversely, A2 astrocytes upregulate neurotrophic or anti-inflammatory genes, promote neuronal survival and growth, and support repair functions, suggesting that they may have a protective role [14,23]. Accordingly, we examined the expression of mRNA markers, such as complement protein 3 (C3) and S100 calcium binding protein A10 (S100A10), in A1 and A2 astrocytes by qRT-PCR. After the intrathecal injection of TNF-α, we found that the mRNA expression of both C3 and S100A10 in the spinal cord of rats was upregulated. The mRNA expression of C3 in the spinal cord of NP rats was significantly downregulated under the intervention of daphnetin. Interestingly, daphnetin had no significant effect on the mRNA of S100A10, a marker of A2 astrocytes. Moreover, our intrathecal intubation operation did not significantly affect the mRNA expression of C3 and S100A10 in the spinal cord of rats. We also performed the immunofluorescence double staining of the A1 and A2 astrocyte markers within the L4–L5 segment of the rat spinal cord and found that both C3 and S100A10 in astrocytes in the dorsal horn of the NP rat spinal cord showed a high expression. After the intrathecal injection of daphnetin, we found that the expression of C3 in the spinal cord of NP rats decreased significantly while S100A10 did not change significantly. However, intrathecal intubation did not significantly affect the polarization of astrocytes in the spinal dorsal horn of rats. See Figure 2.

### 2.4. Daphnetin Inhibits the Polarization of M1 Microglia in the Spinal Cord of NP Rats

**Figure 3:**
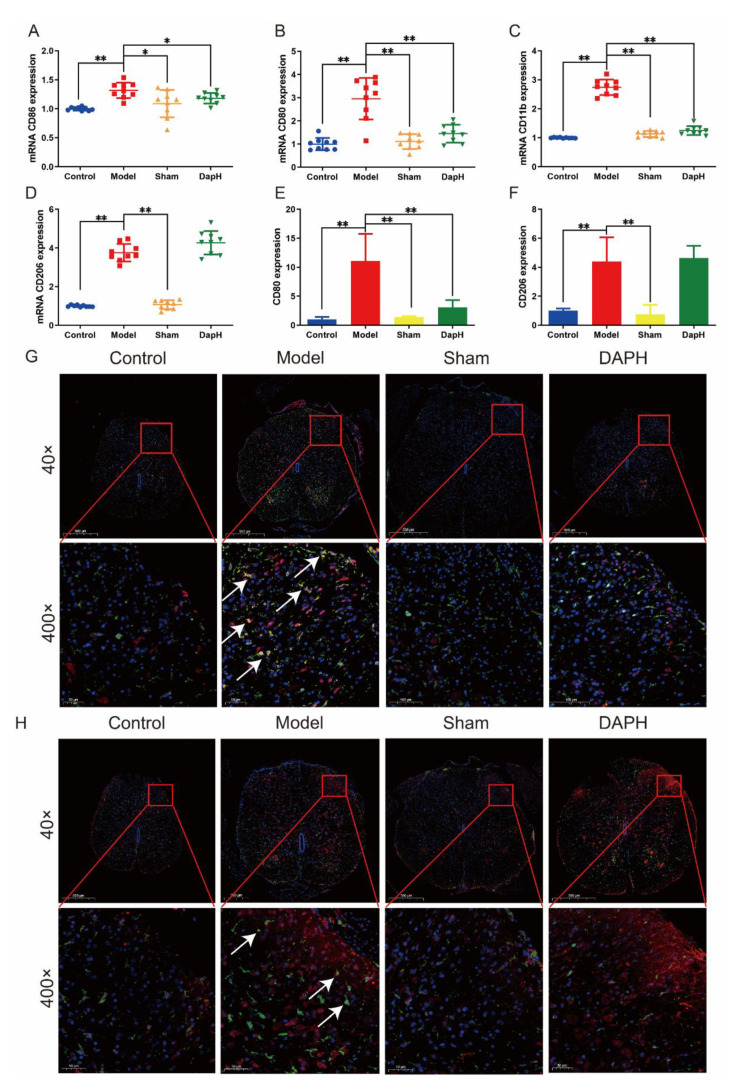
Effect of daphnetin on microglia polarization in the spinal cord of NP rats. (**A**–**C**) mRNA expression of M1 microglial cell markers in the spinal cord (*n* = 3). (**D**) mRNA expression of M2 microglial cell markers in the spinal cord (*n* = 3). (**E**) Immunofluorescence intensity of CD80 in immunofluorescence sections (*n* = 3). (**F**) Immunofluorescence intensity of CD206 in immunofluorescence sections (*n* = 3). (**G**) Immunofluorescence double staining of CD80 (red) and Iba−1 (green) in the spinal cord of NP rats (*n* = 3), the arrows is the overlap of CD80 and Iba−1. (**H**) Immunofluorescence double staining of CD206 (red) and Iba−1 (green) in the spinal cord of NP rats, the arrows is the overlap of CD206 and Iba−1 (*n* = 3). Squares indicates the zoom area. * *p* < 0.05, ** *p* < 0.01. The blue circles, red squares, green and yellow triangles refer to the individual data of the Control, Model, Sham and DAPH groups, respectively.

Under normal conditions, microglia remain stable and protective of neurons. In the NP model induced by peripheral nerve injury, microglia activation is accompanied by an imbalance in polarization, and as nerve injury progresses, microglia in the spinal cord tend towards a single expression of M1 type [10]. The disruption of the dynamic balance between M1 and M2 microglia can lead to a variety of diseases [24], including NP [25,26]. In previous data, we found that daphnetin significantly inhibited the activation of microglia in the rat spinal cord induced by intrathecal injection of TNF-α. For this reason, we detected the mRNA of M1 microglia markers (cluster of differentiation 11b (CD11b), cluster of differentiation 86 (CD86), and cluster of differentiation 80 (CD80)) and M2 microglia markers (cluster of differentiation 206 (CD206)) in the rat spinal cord using the qRT-PCR technique, which was used to investigate the mechanism of action of daphnetin interfering with microglia polarization. As a result, we found that the mRNA of M1 microglia markers (CD11b, CD86, and CD80) and M2 microglia markers (CD206) were significantly upregulated in the rat spinal cord after TNF-α injection. The mRNA of markers CD11b, CD86, and CD80 of M1 microglia were significantly inhibited after the intrathecal injection of daphnetin, in contrast, no significant intervention of the mRNA expression of marker CD206 of M2 microglia was seen with daphnetin. We also performed immunofluorescence double staining of the L4–L5 segment of the rat spinal cord and found that both the CD80 and CD206 of microglia within the dorsal horn of the spinal cord of NP rats showed a high expression. After the intrathecal injection of daphnetin, we found that CD80 in the spinal cord of NP rats decreased significantly, while CD206 remained consistently increased. In contrast, intrathecal cannulation did not show a significant effect on the polarization of microglia in the dorsal horn of the rat spinal cord (see Figure 3).

### 2.5. Daphnetin Inhibits the Expression of CX3CL1/CX3CR1 and CXCL13/CXCR5 Chemokine Pairs in the Spinal Cord of NP Rats

**Figure 4:**
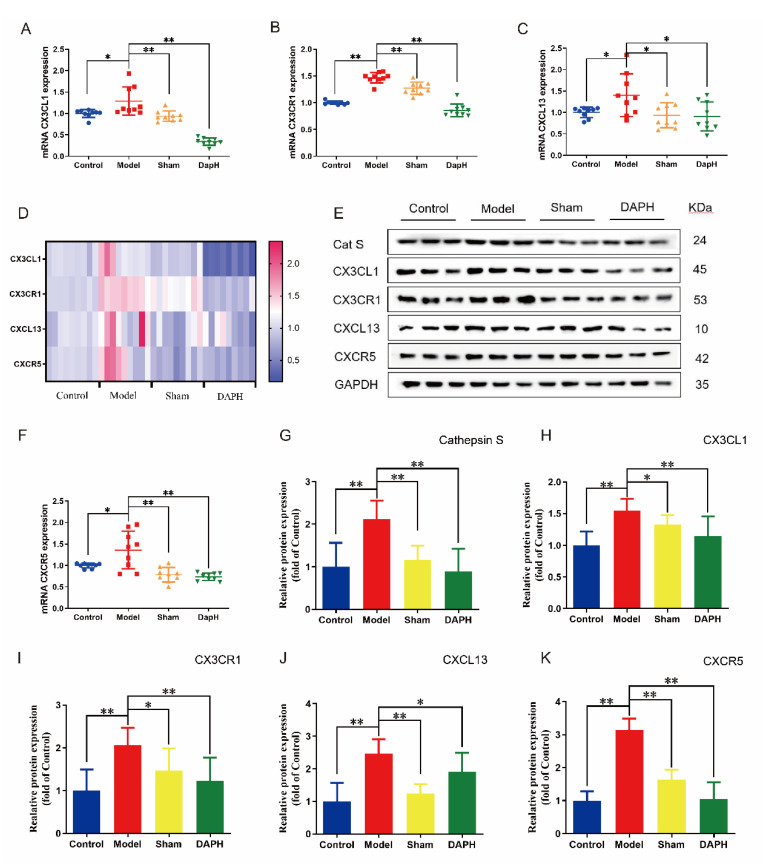
Effect of daphnetin on chemokines in the spinal cord of NP rats. (**A**–**D**,**F**) Expression of chemokines CX3CL1, CXC3R1, CXCL13, and CXCR5 mRNA in the spinal cord of NP rats (*n* = 3). (**E**) Western blot protein bands of chemokines CX3CL1, CXC3R1, CXCL13, and CXCR5 and CatS in the spinal cord of NP rats (*n* = 3). (**G**–**K**) Western blot protein expression of chemokines CX3CL1, CXC3R1, CXCL13, and CXCR5 and CatS in the spinal cord of NP rats (*n* = 3). * *p* < 0.05, ** *p* < 0.01. The blue circles, red squares, green and yellow triangles refer to the individual data of the Control, Model, Sham and DAPH groups, respectively.

C-X3-C motif chemokine 1 (CX3CL1) (Fractalkine, FKN), the only member of the CX3C chemokine family, is abundantly expressed in neurons and is involved in regulating a variety of neurophysiological activities [27]. The characteristics of the CX3CL1 adhesive isomer and chemotactic isomer are mediated by a specific G protein-coupled receptor (CX3CR1), which only exists on microglia [28]. The interaction between the microglia chemokine receptor CX3CR1 and the neuronal ligand CX3CL1 allows for precise and efficient communication between neurons and microglia, and therefore, plays a key role in coordinating many aspects of brain function [28]. Cathepsin S (CatS) cleaves the CX3CL1 chemokine structural domain to neuronal membranes and precisely regulates CX3CL1 expression upstream, which in turn is involved in NP maintenance [29,30]. In this experiment, we examined the mRNA and protein expression of CX3CL1 and CX3CR1 and CatS protein expression in the spinal cord of NP rats and found that, 24 h after the intrathecal injection of TNF-α, the mRNA and protein of CX3CL1 and CX3CR1 were overexpressed in the spinal cord of NP rats and CatS protein expression was upregulated. The mRNA and protein expression of CX3CL1 and CX3CR1 were suppressed and CatS protein expression was downregulated after the intrathecal injection of daphnetin. Details can be seen in Figure 4A,B,D,E,G–I.

C-X-C motif chemokine 13 (CXCL13), also known as B-lymphocyte chemokine, was originally identified in the stromal cells of B-cell follicles [31]. It has been shown that CXCL13 is upregulated in the spinal nerve ligation (SNL) model and induces the NP through the activation of astrocytes in the spinal cord [32]. C-X-C chemokine receptor type 5 (CXCR5), as the receptor of CXCL13, exists in astrocytes or B-lymphocyte and T-lymphocyte subtypes in blood, lymphoid tissue, and cerebrospinal fluid [33]. CXCL13 mediates NP through CXCR5-dependent astrocyte activation [32]. We examined the mRNA and protein expression of CXCL13/CXCR5 chemokine pairs in the spinal cord of rats in the model group and found that the rats showed high mRNA and protein expression of CXCL13/CXCR5 in the NP state. CXCL13/CXCR5 expression was substantially attenuated when intrathecal daphnetin was injected (see Figure 4C–F,J,K).

### 2.6. Intervention of Daphnetin on the Polarization of HMC3 Cells

**Figure 5:**
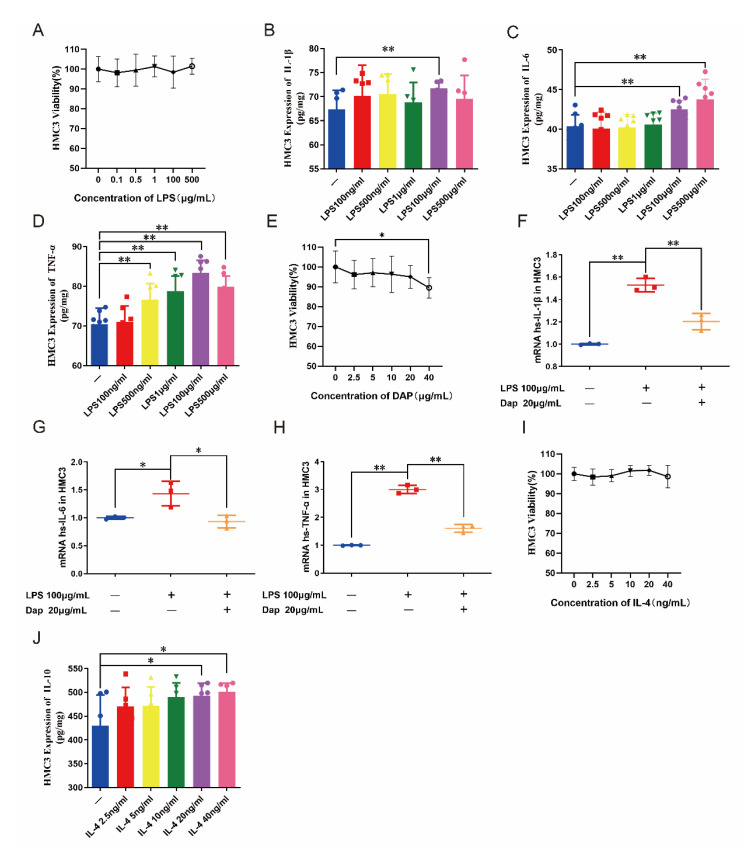
Effect of daphnetin on HMC3 cells. (**A**) Effect of different concentrations of LPS on the cell viability of HMC3 (*n* = 6). (**B**–**D**) Effect of different concentrations of LPS on the expression of inflammatory factors in HMC3 cells (*n* = 6). (**E**) Effect of different concentrations of daphnetin on the cell viability of HMC3 cells (*n* = 6). (**F**–**H**) Effect of daphnetin on inflammatory factors in LPS−induced HMC3 cells (*n* = 6). (**I**) Effect of different concentrations of IL−4 on the cell viability of HMC3 (*n* = 6). (**J**) Effect of different concentrations of IL-4 on the expression of inflammatory factors in HMC3 cells (*n* = 6). * *p* < 0.05, ** *p* < 0.01. Blue circles, red squares, green triangles, yellow triangles, purple and pink circles refer to the individual data of each group, respectively.

Lipopolysaccharide (LPS) induces microglial cell activation in vitro and polarization toward M1 microglia [10]. To assess the effect of LPS on human microglia cell 3 (HMC3) cells, we treated HMC3 cells for 24 h using different concentrations of LPS (100, 500 ng/mL, 1, 100, and 500 μg/mL). The cell counting kit-8 (CCK8) assay of HMC3 cell viability revealed that none of the concentrations of LPS affected the cellular activity of HMC3 cells. The enzyme-linked immunosorbent assay (ELISA) kit assay revealed that LPS induced HMC3 cells to express IL-6 and TNF-α in a concentration-dependent manner, but IL-1β only displayed high expression at a concentration of 100 μg/mL, so we chose a modeling concentration of 100 μg/mL for the subsequent experiments. Similarly, we examined the effect of different concentrations of daphnetin (2.5, 5, 10, 20, and 40 μg/mL) on the cell viability of HMC3 using CCK8 to determine the concentration of daphnetin administration. The cell viability of HMC3 was found to be inhibited with increasing concentrations of daphnetin. Among them, the cell viability of HMC3 under the effect of 20 μg/mL of daphnetin was not significantly different from that of normal HMC3, so we selected this concentration of daphnetin to intervene in HMC3 cells in subsequent experiments. After the pretreatment of HMC3 cells with 20 μg/mL daphnetin for 24 h, 100 μg/mL LPS was added to stimulate HMC3 cells for 24 h. The expression of inflammatory factor mRNA in HMC3 cells was detected by the qRT-PCR technique. It was found that LPS was able to induce the expression of IL-1β, IL-6, and TNF-α mRNA in HMC3 cells, and the IL-1β, IL-6, and TNF-α mRNA produced by HMC3 cells were effectively inhibited after pretreatment with daphnetin. It is suggested that daphnetin can inhibit the secretion of inflammatory factors by HMC3 cells under LPS stimulation (see Figure 5A–H).

IL-4 selectively stimulates microglia to polarize to M2 microglia for neuroprotective effects [34]. We treated HMC3 cells with different concentrations of IL-4 (2.5, 5, 10, 20, and 40 μg/mL) for 24 h. Using CCK8 to detect the effect of IL-4 on the cell viability of HMC3, we found that all concentrations of IL-4 had no effect on the cell viability of HMC3. Therefore, we measured the concentration of IL-10 in HMC3 cell medium using ELISA kits to evaluate the effect of different concentrations of IL-4 on HMC3 cells. The expression of IL-10 in HMC3 cells was found to increase gradually with the increase in IL-4 concentration. With no effect on the cell viability of HMC3, we finally selected a concentration of 40 μg/mL IL-4 for subsequent experiments. After the pretreatment of HMC3 cells with 20 μg/mL daphnetin, HMC3 cells were stimulated with 40 μg/mL IL-4 for 24 h, and the expression of anti-inflammatory factors in HMC3 cells was detected using the qRT-PCR technique to evaluate the intervention of daphnetin on HMC3 cell activation. It was found that IL-4 was able to induce the production of mRNA of IL-10 in HMC3 cells. After pretreatment with daphnetin, it was able to stimulate the production of excessive mRNA of IL-10 in HMC3, which was able to promote the production of anti-inflammatory factors in HMC3 cells (see Figure 5I,J).

**Figure 6:**
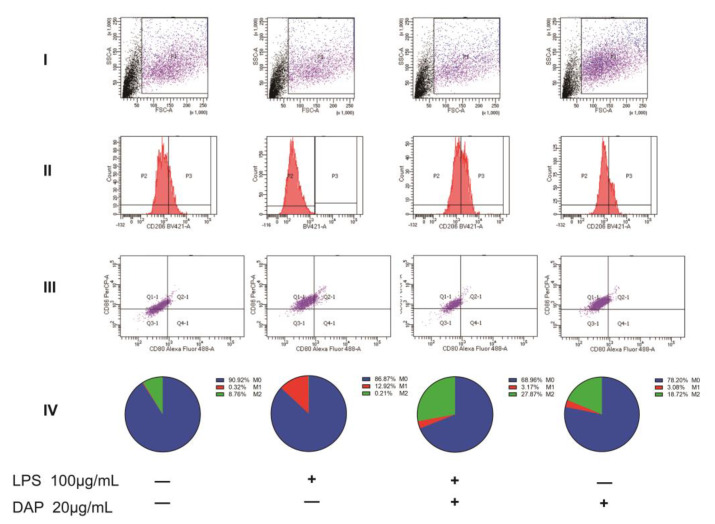
Effect of daphnetin on the LPS−induced polarization of HMC3 cells. (**I**) The range of detection circled by flow cytometry. (**II**) Negative region P2 and positive region P3 of CD206. (**III**) Expression of CD80 and CD86 in cells within the P2 region. (**IV**) Percentage expression of M0, M1, and M2 within the samples by flow cytometry (*n* = 3).

**Figure 7:**
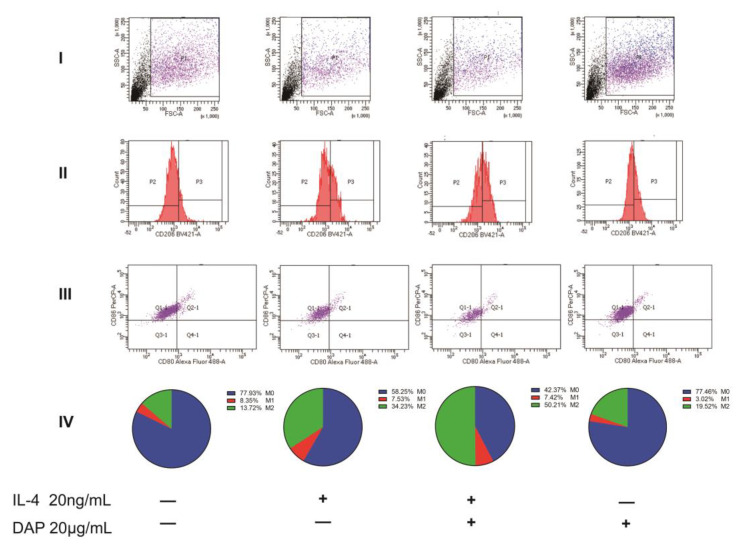
Effect of daphnetin on the IL−4−induced polarization of HMC3 cells. (**I**) The range of detection circled by flow cytometry. (**II**) Negative region P2 and positive region P3 of CD206. (**III**) Expression of CD80 and CD86 in cells within the P2 region. (**IV**) Percentage expression of M0, M1, and M2 within the samples by flow cytometry (*n* = 3).

Using flow cytometry, we examined the expression of CD80, CD86, and CD206 in HMC3 cells. We collected cells in the P1 region and used markers for CD206 to distinguish between M0/M1 and M2 and markers for CD80 and CD86 to distinguish between M0 and M1. Of these, M0 cells expressed only CD86 and M1 cells expressed CD80 and CD86 [35]. In the results, we can see that the untreated HMC3 cells were 8.67% positive for CD206 and showed more polarization in favor of M2. Under the induction of LPS, the CD206 positivity rate of HMC3 cells was 0.21%, and M2 cells almost ceased to express; in contrast, the positive expression rate of M1 reached 12.1%, and the cells as a whole tended to polarize toward M1 cells. After the administration of 20 μg/mL of daphnetin, we found that the positive expression rate of M1 decreased to 3.17%, while the positive expression rate of M2 reached 27.78%, indicating that daphnetin could inhibit the polarization of HMC3 cells toward M1 at the same time and induce the polarization of M1 cells into M2 cells. Under the induction of IL-4, the positive rate of M2 in HMC3 cells was 34.23%, but the positive rate of M1 cells was 7.53% compared with the M1 positive rate of 8.35% in untreated HMC3 cells indicating no significant difference and suggesting that IL-4 was able to induce the polarization of M0 to M2 cells in HMC3 cells but had no effect on M1 cells. After the administration of 20 μg/mL daphnetin, we found a positive expression rate of 50.21% in M2 cells, indicating that daphnetin is able to synergistically induce HMC3 polarization toward M2 cells with IL-4. As for the effect of daphnetin on HMC3 cells, we found that daphnetin could induce the polarization of HMC3 cells to the M2 direction to some extent. All the above data indicate that daphnetin has a strong anti-inflammatory effect and is able to inhibit the polarization of HMC3 cells to M1 cells while inducing the conversion of M1 cells to M2 cells (see Figure 6 and Figure 7).

### 2.7. Intervention of Daphnetin on Polarization of U251 Cells

**Figure 8:**
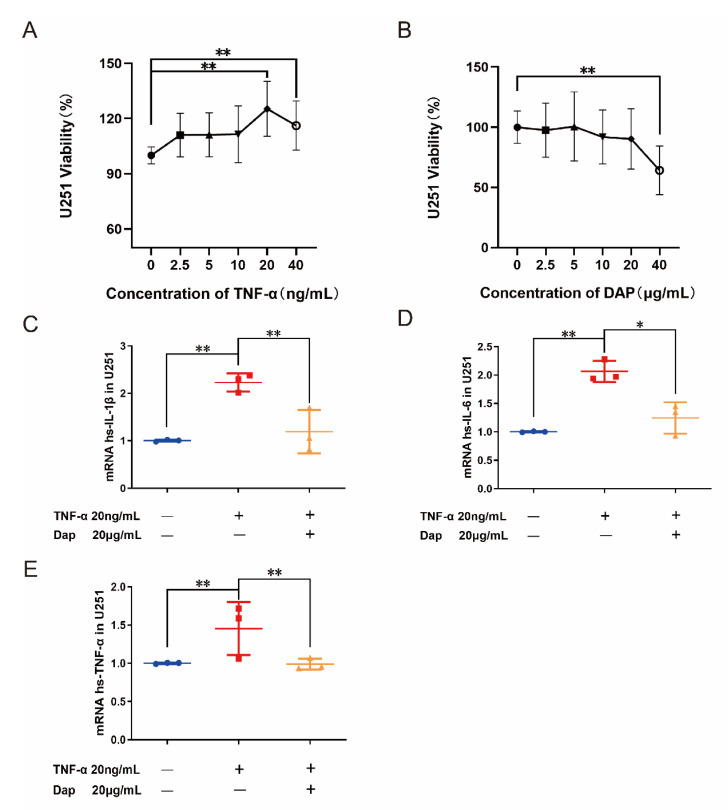
Effect of daphnetin on U251 cells. (**A**) Effect of different concentrations of TNF−α on cell viability of U251 (*n* = 6). (**B**) Effect of different concentrations of daphnetin on cell viability of U251 (*n* = 6). (**C**–**E**) Effect of daphnetin on IL−4−induced inflammatory factors in U251 cells (*n* = 6). * *p* < 0.05, ** *p* < 0.01. Blue circles, red squares and yellow triangles refer to the individual data of each group, respectively.

TNF-α activates the nuclear factor kappa-B (NF-κB) pathway in astrocytes and causes edema in astrocytes [36]. The U251 cell line has been studied as an astrocyte subject in several papers [37,38]. To evaluate the effect of TNF-α on U251 cells, we treated U251 cells for 24 h using different concentrations of TNF-α (2.5, 5, 10, 20, and 40 ng/mL). The CCK8 assay of U251 cell viability revealed that TNF-α was able to effectively increase the cell viability of U251, where a significant difference appeared between the doses of 20 and 40 ng/mL, according to which we chose the concentration of 20 ng/mL for the subsequent modeling. We also examined the effect of different concentrations of daphnetin (2.5, 5, 10, 20, and 40 μg/mL) on the cell viability of U251, using CCK8 to determine the concentration of daphnetin administration. We found that 40 μg/mL of daphnetin effectively inhibited the cell viability of U251, while 20 μg/mL had no significant effect on cell viability, so we chose 20 μg/mL of daphnetin for the subsequent experiments. After the pretreatment of U251 cells using 20 μg/mL daphnetin, 10 ng/mL TNF-α was added to stimulate U251 cells for 24 h. The expression of mRNA of inflammatory factors in U251 cells was detected by the qRT-PCR technique. It was found that TNF-α was able to induce mRNA of IL-1β, IL-6, and TNF-α production in U251 cells, and the mRNA of IL-1β, IL-6, and TNF-α produced by HMC3 was effectively inhibited after pretreatment with daphnetin, suggesting that daphnetin was able to inhibit the TNF-α-induced secretion of inflammatory factors in U251 cells (see Figure 8).

**Figure 9:**
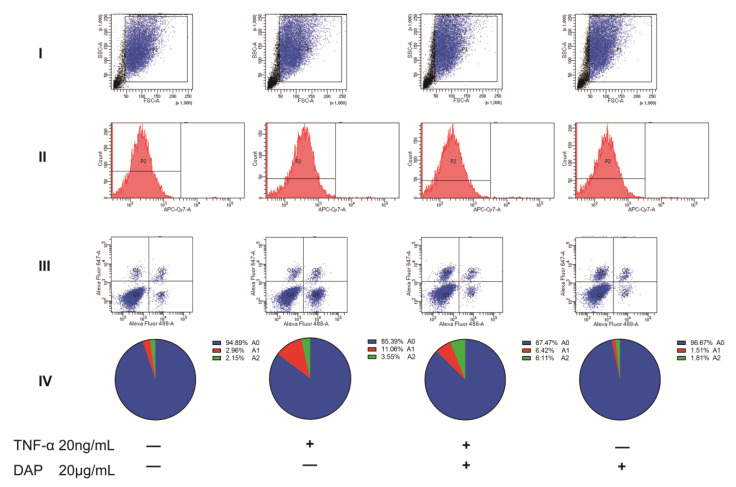
Effect of daphnetin on the TNF−α−induced polarization of U251 cells. (**I**) The range of cells circled by flow cytometry. (**II**) Negative region P2 and positive region P3 of Zombie NIR. (**III**) Expression of C3 and S100A10 in cells within the P2 region. (**IV**) Percentage expression of A0, A1, and A2 within the samples by flow cytometry (*n* = 6).

Using flow cytometry, we examined the expression of C3 and S100A10 in U251 cells, and Zombie NIR was used to show the survival status of U251 cells. We collected cells in the P1 region and selected Zombie NIR-negative cells for C3 and S100A10 sorting [39]. In the results, we can see that the positive expression of A1 and A2 astrocytes in U251 cells was 2.96% and 2.15%, respectively. Under the induction of TNF-α, the positive expression rate of A1 astrocytes reached 11%, while the expression rate of A2 astrocytes was 3.55%. After pretreatment with daphnetin, the positive expression of A1 in U251 cells decreased to 6.42%, while the positive expression of A2 was upregulated to 6.11%. The above data suggest that daphnetin can effectively interfere with the TNF-α-induced polarization of U251 cells to A1 astrocytes while inducing the conversion of A1 astrocytes to A2 astrocytes. In contrast, when daphnetin interfered with U251 cells, the positive expression rate of A1 and A2 astrocytes among them was 1.51% and 1.81%, respectively, indicating that daphnetin did not show a significant intervention on the polarization of normal cultured U251 cells (see Figure 9).

## 3. Discussion

Peripheral nerve injury can activate glial cells in the spinal cord, which release inflammatory mediators and lead to central sensitization, resulting in the formation of NP [40]. The upregulation of multiple inflammatory factors (IL-1β, IL-6, interleukin-17 (IL-17), and TNF-α) can be found in the model of NP [14]. The intrathecal injection of TNF-α can directly induce hyperalgesia in rodents [41,42]. Therefore, we used an animal model of NP induction through the intrathecal injection of TNF-α in the present study. We observed a significant downregulation in both TWT and MWT in rats after the intrathecal injection of daphnetin for 1 h. This effect lasted for 24 h. We reasonably speculated that the intrathecal injection of daphnetin was able to effectively improve NP in rats. The typical inflammatory pathway NF-κB and the release of inflammatory factors mediated by inhibiting the apoptosis proteins (IAPS) and cyclooxygenase-2 (COX-2) were also inhibited by daphnetin [16]. Some scholars pointed out that daphnetin was able to effectively inhibit the activation of glial cells in the spinal cord in inflammatory pain, which is related to its inhibition of the NF-κB pathway in the spinal cord [43]. Consistent with the findings of Yang et al., we also observed that daphnetin inhibited the activation of microglia, astrocytes, and neurons induced by TNF-α in the rat spinal cord and subsequently reduced the mRNA expression of IL-1β, IL-6, and TNF-α.

Chemokines play a key role in the activation and infiltration of macrophages and glial cells under NP conditions [8,44]. In the NP state, astrocytes can receive CXCL13 released by activated neurons in addition to the excessive release of inflammatory factors and chemokine C-X-C motif ligand 1 (CXCL1), which further releases thrombospondin 4 (TSP4) for the regulation of synapse formation, synaptic plasticity, and nociceptive hypersensitivity [45,46]. It has been documented that blocking CXCR5 function in spinal astrocytes reduces allodynia in chronic pain conditions [47]. In this subsection of the experiment, similarly, we observed that daphnetin effectively inhibited the expression of CXCL13/CXCR5 chemokines induced by TNF-α on mRNA and protein. Therefore, we reasonably speculate that daphnetin inhibits TNF-α-induced astrocyte activation and further reduces astrocyte activation by reducing CXCR5 receptor expression and neuronal CXCL13 release, thus cutting off the crosstalk between astrocytes and neurons via chemokine pairs.

The neuronal production of CX3CL1 in the central nervous system is essential for neuron-glial signaling [48]. Neural tissue injury leads to CatS cleavage and the activation of chemokine CX3CL1, which in turn stimulates CX3CR1 receptors in microglia leading to microglia activation [29]. Microglia are critical for NP, and the upregulation of CX3CR1 is seen in the CCI model and peaks at day 10, but hyperalgesia responses induced by nerve injury are substantially reduced after CX3CR1-neutralizing antibody injection [49,50]. Blocking CatS, knocking out CX3CR1, or inhibiting the expression of CX3CL1 in neurons can prevent NP induced by peripheral nerve injury [51]. After the intrathecal injection of daphnetin, we found that the protein expression of CatS was suppressed, as were the mRNA and protein expression of CX3CL1 and CX3CR1. While inhibiting neuronal activation, daphnetin reduced CX3CL1 expression and inhibited CX3CR1 receptor activity in microglia, further interfering with the process of microglia activation induced by activated neurons via CX3CL1 and cutting off the crosstalk between neurons and microglia via chemokine pairs.

In the NP model, astrocytes in the dorsal horn of the spinal cord are activated, prompting the transformation of resting astrocytes into A1 astrocytes, which may be involved in the maintenance of NP [14]. C3, a central molecule in the complement signaling pathway, is a specific marker of A1 astrocytes and is often identified as an important inflammatory factor [14]. A1 astrocytes directly modulate neurons by releasing C3 and activating receptors on neurons [23]. The inhibition of A1 astrocyte production and the promotion of A2 astrocyte production help to alleviate allodynia in rats [37]. In this experiment, we observed that daphnetin was able to reduce the mRNA expression of C3 in the spinal cord of NP rats and decrease the number of A1 astrocytes in the spinal cord, which is essential for daphnetin to improve NP in rats. The results of immunofluorescence double staining could also demonstrate that the C3 protein expression of astrocytes in the dorsal horn of the spinal cord was substantially reduced in response to daphnetin. As seen from the qRT-PCR and immunofluorescence results, there was no excessive modulation of S100A10, a marker of A2 astrocytes, by daphnetin, suggesting that daphnetin has no significant intervention on A2 astrocytes. In the flow cytometry assay we found that daphnetin was able to inhibit the TNF-α-induced polarization of U251 cells toward A1 astrocytes, which was consistent with our in vivo experimental results. We found that daphnetin was able to induce the conversion of A1 astrocytes to A2 astrocytes and promote the polarization of A0 astrocytes to A2 astrocytes by flow cytometry data.

Activated microglia have been reported to induce their polarization towards the M1 type by secreting pro-inflammatory factors (IL-1β, IL-6, and TNF-α) while activating multiple signaling pathways to enhance neuronal excitability [11], while IL-10 induces their polarization towards the M2 type by release [52]. Our experiments revealed a substantial decrease in the mRNA expression of M1 microglia markers (CD11b, CD86, and CD80) in the rat spinal cord after the intrathecal injection of daphnetin, while no significant intervention was seen for the mRNA marker of M2 microglia (CD206). Combined with the immunofluorescence results, we found that daphnetin was able to effectively inhibit the polarization of microglia to M1 microglia, while for polarization to M2 microglia, daphnetin failed to play a significant intervention role. It has been reported that LPS-induced IL-1β expression by HMC3 peaks at 4 h and declines dramatically at 24 h, and it was noted that no significant positive correlation was seen between IL-1β expression and LPS concentration [53]. In in vitro experiments, we likewise observed that high concentrations of LPS failed to cause HMC3 cells to express more IL-1β, but this did not affect our ability to determine the concentration of LPS required. In the results of flow cytometry, we observed the polarization of HMC3 cells towards M1 and M2 directions induced by LPS and IL-4, respectively. Daphnetin was able to prevent the polarization of microglia toward M1 while inducing the conversion of M1 microglia to M2 microglia and was also able to induce the polarization of M0 microglia to M2 microglia.

It has been shown that in the treatment of fibromyalgia, daphnetin was able to reduce inflammatory factors and glutathione (GSH) in the serum of mice with fibromyalgia and also reduce monoamine oxidase-A (MAO-A) in the brains of mice, which illustrates the protective effect of ryanodine on neurons from the perspective of neurotransmitter depletion and oxidative stress [54]. This study mainly focuses on the spinal cords of rats, specifically the crosstalk between glial cells and neurons. However, research on the oxidative stress of neurons in the brain is still limited, which is also a drawback of our research. For future research directions, we will focus on the role of daphnetin in the intervention of neurons in the brain with the aim of examining the mechanism of daphnetin in the intervention of neuropathic pain from a more comprehensive perspective.

## 4. Materials and Methods

### 4.1. NP Modeling

The experimental protocols of the animal experiments involved were reviewed and approved by the Medical and Laboratory Animal Ethics Committee of Beijing University of Chinese Medicine (NO: BUM4-202103201-1078). Eight-week-old healthy male Sprague Dawley (SD) rats were provided by Beijing SPF Laboratory Animal Technology Co., Ltd. (Beijing, China), Animal License No. SCXK2020-0033. All rats were housed under standardized conditions (in a 12 h light/12 h dark cycle, constant temperature (23 ± 1 °C), and relative humidity (60 ± 5%)) and allowed free access to water and food. All the rats were acclimated to their environment for one week before the experiment.

An EP-10 catheter (302021, Smiths Medical, London, UK) was placed into the rat subdural space at L4–L5, according to the method outlined in the literature [55]. On the fifth postoperative day, 25 μL of lidocaine hydrochloride injection (XB19H26, Hualu Pharmaceutical Co., Ltd., Shandong, China) was injected through an EP catheter, and if the lower limbs of the rats were immediately paralyzed, the position of the catheter was proven to be at the L4–L5 position in the rats and the rats with successful cannulation were screened for subsequent experiments. The success rate was about 70% when lidocaine was used to verify that the catheter was correctly positioned. Successfully intubated rats were selected for the intrathecal injection of TNF-α (400-14, PeproTech, Suzhou, China) for the induction of an NP model [56]. The injection concentration of TNF-α was 0.5 μg/mL, the injection volume was 25 μL, and the total amount of TNF-α injected was 7.5 ng/rat.

### 4.2. Grouping and Drug Administration

Rats with successful intrathecal cannulation were randomly divided into three groups (*n* = 27), namely, a sham group (intrathecal injection of saline, 25 μL/rat), a model group (intrathecal injection of TNF-α and saline, 25 μL/rat), and a Daph group (intrathecal injection of TNF-α and daphnetin (0.5 mg/mL, (R18J9F5311, Yuanye Biotechnology Co., Ltd., Shanghai, China, HPLC ≥ 98%), 25 μL/rat). In addition, nine rats were set up as a control group (no treatment). The intrathecal injection of the drug began 1 h after modeling and was performed once a day for 3 days.

### 4.3. Behavioral Testing

According to the literature [57,58], Von Frey hair (NC12775, Yuyan Scientific Instrument Co., Ltd., Shanghai, China) and a cold/hot disc pain meter (PE34, IITC, Woodland Hills, CA, USA) were used to detect the MWT and TWT in NP rats, respectively.

### 4.4. Immunofluorescence Staining

The L4–L5 spinal cord tissues of three rats in each group were selected and fixed in 10% neutral formalin, dehydrated in ethanol, embedded in wax, and then sectioned at a thickness of 4 µm. Spinal cord sections were dewaxed and incubated overnight at 4 °C with CD80 antibody (1:100, bs-2211R, Bioss, Beijing, China) and Iba-1 antibody (1:100, ab283319, Abcam, Cambridge, UK); CD206 antibody (1:100, DF4149, Affinity, Suzhou, China) and Iba-1 antibody (1:100, ab283319, Abcam, Cambridge, UK) were mixed and incubated overnight at 4 °C; C3 antibody (1:100, DF13224, Affinity, Suzhou, China) and GFAP antibody (1:100, ab279290, Abcam, Cambridge, UK) were mixed and incubated overnight at 4 °C; S100A10 antibody (1:100, bs-8503, Bioss, Beijing, China) and GFAP antibody (1:100, ab279290, Abcam, Cambridge, UK) were mixed and incubated overnight at 4 °C. Goat anti-rabbit IgG H&L (Alexa Flour 488,1:200, ab150077, Abcam, Cambridge, UK) and goat anti-mouse IgG H&L (Alexa Flour 647,1:200, ab150115, Abcam, Cambridge, UK) were used as secondary antibodies. Tissue sections were immunofluorescently stained and then observed under a laser confocal microscope (SP8, Leica, Wetzlar, Germany) and photographed. Fluorescence quantitative analysis was performed by Image J software.

### 4.5. Real-Time Fluorescence Quantitative PCR Detection of Relevant mRNA Content

Three L4–L5 spinal cords from each group were selected, and total RNA was extracted using Trizol (94604, Ambion, Austin, TX, USA) and reverse transcribed into single-stranded cDNA using the gDNA purge (E047-01B, NovoScript^®^, Shanghai, China) kit. The relevant primer gene content in spinal cord tissues was detected using SYBR Green Master Mix (E096-01B, NovoScript^®^, Shanghai, China) dye in a qRT-PCR instrument (ABI, StepOnePlus, Foster City, CA, USA). The primer design is shown in Table 1.

### 4.6. Western Blot to Determine Protein Expression

We extracted total protein from the L4–L5 spinal cords of three rats selected in each group by grinding in liquid nitrogen and adding to RAPI lysate (P0013C, Beyotime, Haimen, China) containing protease and phosphoproteinase inhibitors (20210401, NCM, Tianjin, China). After the detection of protein concentration by the BCA kit (120320210419, Beyotime, Haimen, China), 10 μg of protein were separated on 10% SDS-PAGE and transferred to PVDF membrane (R1DB92455, Millipore, Darmstadt, Germany). The membranes were blocked at room temperature in 5% skimmed milk for 1.5 h and then incubated with Cathepsin S (1:1000, bs-8558R, Bioss, Beijing, China), CX3CL1 (1:1000, A14198, ABclonal, Woburn, MA, USA), CX3CR1 (1:1000, bs-1728R, Bioss, Beijing, China), CXCL13 (1:1000, bs-4509R, Bioss, Beijing, China), CXCR5 (1:1000, bs-3598R, Bioss, Beijing, China), and GAPDH (1:10,000, 60004-1-Ig, Proteintech, Rosemont, IL, USA) antibodies overnight at 4 °C. The membranes were washed three times with TBST (T1081, Solarbio, Beijing, China) and incubated with goat anti-rabbit IgG HRP (1:5000, S0101, LABLEAD, Beijing, China) or goat anti-mouse IgG HRP (1:5000, S0100, LABLEAD, Beijing, China) at room temperature for 1 h. The ECL hypersensitivity kit (P2200, NCM, Suzhou, China) was developed. The protein strip signal was detected using an Amersham ECL system (Amersham Imager 680, Pittsburgh, PA, USA). The protein bands were quantified using the Image J software. All the Western blot strip data were obtained from three independent replicates.

### 4.7. Cell Culture

The HMC3 cell line and U251 cell line were purchased from Pricella (Wuhan, China). Cells were grown freely in DMEM medium (812208, Gibco, Shanghai, China) supplemented with 10% fetal bovine serum (FBS) (35-076-CV, Corning cellgro, New York, NY, USA), 100 IU/mL penicillin, and 10 μg/mL streptomycin (15140-122, Gibco, New York, NY, USA) and maintained in a 37 °C, 5% CO_2_ humidified incubator (311, Thermo Fisher, Waltham, MA, USA). HMC3 cells and U251 cells were passaged to a logarithmic growth phase for subsequent experiments, respectively.

### 4.8. Cell Viability Testing

HMC3 cells were cultured to a logarithmic growth phase and inoculated in 96-well plates at a density of 5 × 10^3^ cells/well with 100 and 500 ng/mL and 1, 100, and 500 μg/mL of LPS, (L2880-10MG. Sigma, St. Louis, MO, USA) or 2.5, 5, 10, 20, and 40 ng/mL of IL-4 (AF-200-04-5, PeproTech, Suzhou, China) or 2.5, 5, 10, 20, and 40 μg/mL of Dap and were incubated for 24 h. After that, 10 μL CCK8 (CK04, DOJINDO, Munich, Germany) was added to each well and incubated for 1 h. The cell viability of HMC3 cells was detected at 450 nm of the microplate reader (Epoch, BioTek, Winooski, VT, USA).

U251 cells were cultured to a logarithmic growth phase, inoculated in 96-well plates at a density of 5 × 10^3^ cells/well, and incubated with 2.5, 5, 10, 20, and 40 ng/mL of TNF-α (300-01A-10, PeproTech, Suzhou, China) or 2.5, 5, 10, 20, and 40 μg/mL of Dap for 24 h. After incubation, 10 μL CCK8 was added to each well for 1 h. The viability of U251 cells was detected at 450 nm of the microplate reader.

### 4.9. Biochemical Testing

The supernatants of HMC3 cells were collected after 24 h of stimulation with each concentration of LPS, and the levels of IL-1β (ML058097, Mlbio, Shanghai, China), IL-6 (ML064303, Mlbio, Shanghai, China) and TNF-α (ML064299, Mlbio, Shanghai, China) were detected using ELISA kits. The supernatants of HMC3 cells were collected 24 h after stimulation with each concentration of IL-4, and the content of IL-10 (YJ064299, Mlbio, Shanghai, China) was detected using ELISA kits.

### 4.10. Flow Cytometry

HMC3 cells in the logarithmic growth phase were inoculated in 6-well plates (2 × 10^5^ cells/well). After treatment, trypsin (25200-056, Gibco, Shanghai, China) was used for digestion, PBS (AG29574691, Cytiva, Marlborough, MA, USA) was used for washing, and 5 μL of CD80-AF488 (305214, Biolegend, San Diego, CA, USA), CD86-PE-Cy5.5 (393113, Biolegend, San Diego, CA, USA) and CD206-BV421 (321126, Biolegend, San Diego, CA, USA) antibodies were incubated with the cells at 4 °C for 40 min, and the cells were resuspended after antibody cleaning. The polarization states of HMC3 cells were assessed by measuring M1 (CD80^+^/CD86^+^) and M2 (CD206^+^) specific markers by flow cytometry (FACSCanto II, BD, NYC, USA). All the flow cytometry data were obtained from three independent replicates.

U251 cells in the logarithmic growth phase were inoculated in 6-well plates (2 × 10^5^ cells/well). At the end of treatment, after digestion with trypsin and washing with PBS, the flow cytometric antibody Zombie NIR (423105, Biolegend, San Diego, CA, USA) was added and incubated for 15 min at 4 °C. After washing with PBS, 250 mL of Fixation Buffer (420801, Biolegend, San Diego, CA, USA) was added and incubated for 20 min at room temperature and protected from light. After washing twice with Intracellular Staining Perm Wash Buffer (421002, Biolegend, San Diego, CA, USA), 1 μL of C3-AF488 (ab196458, Abcam, Cambridge, UK) and 1 μL of S100A10 (5529, CST, Boston, MA, USA) were added and incubated for 40 min at 4 °C, followed by 1 μL of Goat Anti-Mouse IgG H&L/Cy5 (bs-0296G-Cy5, Bioss, Beijing, China), incubated for 30 min at 4 °C and resuspended with PBS.

### 4.11. Statistical Analysis

SAS 8.2 was used for statistical processing, and all the data are expressed as the mean ± standard deviation (S.D.). When the sample size does not conform to a normal distribution, a nonparametric test is used. When the data conformed to the normal distribution, the two-way Student’s *t*-test was used for comparison between two groups (un-paired), one-way ANOVA was used for three or more groups, and the LSD method was used to compare the groups. Behavioral data were analyzed by the repeated measures ANOVA method. Significant differences were set at *p* < 0.05.

## 5. Conclusions

Daphnetin inhibited the expression of inflammatory factors IL-1β, IL-6, and TNF-α in the spinal cord of NP rats and suppressed the activation of microglia, astrocytes, and neurons in the spinal cord of NP rats. Daphnetin blocked astrocyte–neuron as well as neuron–microglia crosstalk by interfering with the expression of CXCL13/CXCR5 and CX3CL1/CX3CR1. Meanwhile, daphnetin was able to promote the polarization of M1 microglia and A1 astrocytes toward M2 microglia and A2 astrocytes, respectively, further weakening the central sensitization induced by glial cell polarization, thus reducing the nociceptive sensitization in NP rats.

## Figures and Tables

**Table 1:** Primer sequences.

Primer	Forward	Reverse
*Gfap*	GAGTGGTATCGGTCCAAGTT	CTCAAGGTCGCAGGTCAA
*Iba-1*	ATGAGCCAGAGCAAGGATT	GCATTCGCTTCAAGGACA
*C-fos*	TGTGACCTCCCTGGACTTG	CACTGGGCCTAGATGATGC
*Il-1β*	GGCAACTGTCCCTGAACT	TCCACAGCCACAATGAGT
*Il-6*	TCTCTCCGCAAGAGACTTCC	CCGGACTTGTGAAGTAGGGA
*Tnf-α*	ACTCCCAGAAAAGCAAGCAA	CGAGCAGGAATGAGAAGAGG
*Cd86*	ATAGCACTGCATACCTGCCG	AGTCTTTCTGCTGGGTCTGCC
*Cd80*	AGGAGGATCGTACGTGGTGA	GGAGGGTCTTCTGGGGGTTT
*Cd11b*	CTGCCATTACCTCCAACGGT	CTCCCAGGCACCGAAATTCT
*Cd206*	AGTCTGCCTTAACCTGGCAC	AGGCACATCACTTTCCGAGG
*C3*	TGTGGCAGACCCCTATGAGA	TCATTCCTTCTGGCACGACC
*S100a10*	GAAAGGGAGTTCCCTGGGTT	CCCACTTTTCCATCTCGGCA
*Cx3cl1*	TGCACAGCCCAGATCATTCA	CTGCGCTCTCAGATGTAGGAAA
*Cx3cr1*	GGAGCAGGCAGGACAGCAT	CCCTCTCCCTCGCTTGTGTA
*Cxcl13*	CCTTGCAAAAATCAGGCTTCC	CACCTTAGGCTGGTAATGCGTC
*Cxcr5*	CTATTTGCCTTGCCAGAACTCC	CACGAGCATCGGTAGCAGGA
*β-actin*	CTTCCTGGGCATGGAGTCCT	GGA GCA ATGATC TTG ATC TT
*Hs-il-1β*	GGGATAACGAGGCTTATGTGC	AGGTGGAGAGCTTTCAGTTCA
*Hs-il-6*	GACCCAACCACAAATGCCAG	GAGTTGTCATGTCCTGCAGC
*Hs-tnf-α*	TGAGCACTGAAAGCATGATCC	GGAGAAGAGGCTGAGGAACA
*Hs-β-actin*	CTCTTCCAGCCTTCCTTCCT	AGCACTGTGTTGGCGTACAG

## Data Availability

Data are contained within the article.
